# Nanocomposites Prepared from Carbon Nanotubes and the Transition Metal Dichalcogenides WS_2_ and MoS_2_ via Surfactant-Assisted Dispersions as Electrocatalysts for Oxygen Reactions

**DOI:** 10.3390/ma14040896

**Published:** 2021-02-13

**Authors:** Pedro Ferreira, Bárbara Abreu, Cristina Freire, Diana M. Fernandes, Eduardo F. Marques

**Affiliations:** 1 Centro de Investigação em Química, Departamento de Química e Bioquímica, Faculdade de Ciências, Universidade do Porto, 4169-007 Porto, Portugal; up201407953@fc.up.pt (P.F.); barbara.teixeira@fc.up.pt (B.A.); 2 REQUIMTE-LAQV, Departamento de Química e Bioquímica, Faculdade de Ciências, Universidade do Porto, 4169-007 Porto, Portugal; acfreire@fc.up.pt

**Keywords:** nanocomposites, transition metal dichalcogenides, carbon nanotubes, surfactants, non-covalent functionalization, electrocatalysis, oxygen reactions

## Abstract

Fuel cells are emerging devices as clean and renewable energy sources, provided their efficiency is increased. In this work, we prepared nanocomposites based on multiwalled carbon nanotubes (MWNTs) and transition metal dichalcogenides (TMDs), namely WS_2_ and MoS_2_, and evaluated their performance as electrocatalysts for the oxygen evolution reaction (OER) and the oxygen reduction reaction (ORR), relevant to fuel cells. The one- and two-dimensional (1D and 2D) building blocks were initially exfoliated and non-covalently functionalized by surfactants of opposite charge in aqueous media (tetradecyltrimethylammonium bromide, TTAB, for the nanotubes and sodium cholate, SC, for the dichalcogenides), and thereafter, the three-dimensional (3D) MoS_2_@MWNT and WS_2_@MWNT composites were assembled via surfactant-mediated electrostatic interactions. The nanocomposites were characterized by scanning electron microscopy (SEM) and structural differences were found. WS_2_@MWNT and MoS_2_@MWNT show moderate ORR performance with potential onsets of 0.71 and 0.73 V vs. RHE respectively, and diffusion-limiting current densities of −1.87 and −2.74 mA·cm^−2^, respectively. Both materials present, however, better tolerance to methanol crossover when compared to Pt/C and good stability. Regarding OER performance, MoS_2_@MWNT exhibits promising results, with *η*_10_ and *j*_max_ of 0.55 V and 17.96 mA·cm^−2^, respectively. The fabrication method presented here is cost-effective, robust and versatile, opening the doors for the optimization of electrocatalysts’ performance.

## 1. Introduction

Long-lasting and clean energies are vital to the development of future energetic sustainability. The search for electrocatalyst-mediated energy conversion processes has delivered some technologies that, when coupled with renewable energies, are able to convert molecules present in the atmosphere (water, nitrogen or carbon dioxide) in added-value products (hydrogen, hydrocarbons and ammonia). Such processes can be found in many energy storage and conversion devices like metal-air batteries and fuel cells [1,2,3].

The charge and discharge processes of fuel cells and metal-air batteries are dominated by the oxygen-based reactions, oxygen evolution reaction (OER) and oxygen reduction reaction (ORR), respectively. However, the kinetics of these reactions are slow, making them difficult to trigger. Therefore, electrocatalysts are pivotal to increase the rate, efficiency and selectivity of these chemical reactions [1,3,4]. High-performance electrocatalysts should also feature high stability/durability and ORR electrocatalysts resistance to methanol (in direct methanol fuel cells) crossover, something that the current noble metal electrocatalysts do not provide. In addition, due to their scarcity and high price, noble metal catalysts are economically unviable, which promotes the search for more stable and cost-effective alternatives [3,4,5]. Ideally, in reversible fuel cells, electrocatalysts should be bifunctional for ORR and OER and equally high performing. In practice, platinum-based electrocatalysts are deemed the best for ORR, but not sufficiently effective for OER (Pt oxidizes easily at large overpotentials). Likewise, the state-of-the-art OER electrocatalysts (RuO_2_ and IrO_2_) are less effective for ORR [2,3].

In this context, carbon-based nanomaterials emerged as potential alternatives to Pt-based electrocatalysts, and, therefore, have been increasingly investigated. Carbon quantum-dots (CQDs) [6,7], N-doped carbon nanotubes [8,9] and N-doped graphene [10,11] have been reported to have good electrocatalytic behavior towards ORR. Graphene quantum dots, either heteroatom-doped [12] or decorated with non-Pt metals [13,14], have also been described as good ORR electrocatalysts. Many other materials have also exhibited good electrocatalytic behavior toward oxygen reactions, among them polyoxometalates (POMs) [4], perovskites [15], organometallics [16] and spinel family [17] compounds. With respect to transition metal dichalcogenides (TMDs), they have been extensively reported as promising hydrogen evolution reaction (HER) electrocatalysts [18,19,20], and their potential as single materials for oxygen reactions has also been investigated [21,22,23]. Recently, Pumera et al. have studied the ORR electrocatalytic properties of undoped MoS_2_ and WS_2_ and Ti-, V-, Mn- and Fe-doped layered WS_2_ and MoS_2_ [24], demonstrating that not all doping is beneficial. As concerning the use of WS_2_ or MoS_2_ sheets as building blocks of nanocomposite catalysts for ORR, there are only a few reports in the literature [25,26,27].

A promising route for the development and optimization of electrocatalysts is the combination of basic building blocks into new structures, such as one/two-dimensional (1D/2D) composites. In particular, the combination of carbon nanotubes and graphene has been largely studied and was found to result in enhanced properties [28,29,30,31]. Nonetheless, the replacement of graphene with 2D analogues, e.g., TMDs, in such hierarchical structures could unveil improved features [32]. In fact, graphene analogues possess remarkable electronic properties that are tunable according to the number of stacked layers (e.g., bulk 2H-MoS_2_ shows an indirect band gap, but a direct band gap when exfoliated into monolayers) [33,34,35]. Such properties vary relatively weakly with the number of layers as compared to graphene, a material that in contrast requires full exfoliation to monolayers in order to unfold its maximum potential [36].

Some studies regarding the building of CNT/TMD hybrids and their application as electrocatalysts for energy conversion reactions have been reported, mostly dealing with HER [37,38,39,40,41]. Huang et al. fabricated a composite of multiwalled carbon nanotubes (MWNTs) and MoS_2_ using solvothermal synthesis, with the coupling between covalently functionalized nanotubes and MoS_2_ leading to remarkable performance towards HER [37]. A similar type of electrocatalyst was developed by Ahn et al., who applied layer-by-layer assembly to fabricate a MWNT/MoS_2_ thin film, finding the catalytic performance to be dependent on the 1D/2D bilayer number and hence demonstrating the importance of composite architecture for electrocatalytic activity [38]. Notwithstanding their proven applicability for HER, CNT/TMD structures have remained scantly investigated for oxygen reactions, despite revealing potential benefits [42,43]. Recently, Lee et al. found a significant synergistic effect for ORR electrocatalysis from the combination, via hydrothermal method, of functionalized MWNTs and MoS_2_ into a three-dimensional (3D) architecture [42]. In the work of Tiwari et al., WS_2_ and CNTs were interconnected via chemical bonding by the formation of tungsten carbide bonding [43]. These authors showed that WS_2_ sheets on CNT surfaces provide active sites for electrocatalytic activity, while CNTs offer conducting channels and a large surface area, resulting in a bifunctional electrocatalyst for both ORR and OER, with performance comparable to state-of-the-art catalysts (e.g., Pt, RuO_2_).

**Figure 1:**
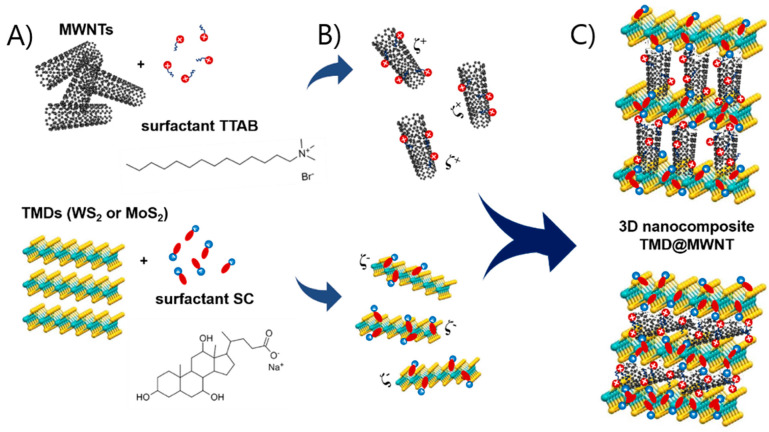
Schematic representation of the assembly process of nanocomposites of multiwalled carbon nanotubes (MWNTs) and transition metal dichalcogenides (TMDs), TMD@MWNT: (**A**) exfoliation and dispersal of the 1D and 2D blocks by cationic surfactant tetradecyltrimethylammonium bromide (TTAB) and anionic surfactant sodium cholate (SC), respectively, (**B**) formation of aqueous dispersions of the charged surfactant-coated particles, and (**C**) assembly of the TMD@MWNT composites via electrostatic attractions, with two possible extremes configurations shown (top, orthogonal layers, and bottom, parallel layers).

In this work, we report the assembly of nanocomposites combining MWNTs and two TMDs, WS_2_ and MoS_2_, and the performance of the obtained WS_2_@MWNT and MoS_2_@MWNT materials as ORR and OER electrocatalysts. The individual building blocks were prepared using surfactants as dispersants and a strictly controlled dispersal procedure in aqueous media [44,45,46,47]. A schematic representation of the process, and its underlying rationale, is shown in Figure 1A–C. As depicted in Figure 1A, the entangled MWNT powder are first exfoliated (by tip sonication) and dispersed using a cationic surfactant (tetradecyltrimethylammonium bromide, TTAB), while the aggregated TMD powder is similarly separated and dispersed using an anionic surfactant (sodium cholate, SC). The surfactants adhere onto the surface of materials essentially by hydrophobic interactions through their hydrocarbon tails, leaving the charged headgroups exposed to the aqueous environment. The obtained dispersions, as shown in Figure 1B, thus consist of positively charged individual MWNTs (or thin bundles thereof), on one side, and negatively charged particles of metal dichalcogenides, on the other side. Both types of surfactant-coated particles possess their electrical double layers and some values of positive and negative zeta potential, respectively. In Figure 1C, mixing of the functionalized blocks in specific proportions leads to the assembly of the composites via electrostatic attractions mediated by the surfactants. In a simplified view, Figure 1C shows two limiting (or idealized) configurations of the resulting materials: in the topmost sketch, the MWNTs are orthogonally placed with respect to the TMD layers (basal planes), and alternate 1D/2D layers are formed; in the bottom one, the MWNTs lie horizontally over the TMD basal planes, forming more tightly bound alternate layers. In reality, it is likely that assorted intermediate configurations will form, such as those having randomly tilted MWNTs or mixed orthogonal/parallel/tilted MWNT layers.

A relevant aspect of this work in relation to the above-mentioned literature is the building of 3D structures resorting to a facile, cost-effective and mild experimental method in aqueous solution via non-covalent functionalization. This methodology aims at fabricating reproducible nanocomposites under controlled and optimizable conditions. After their formation, the designed materials were structurally characterized using SEM and their individual performance as ORR and OER electrocatalysts was assessed. For this, we used both cyclic (CV) and linear sweep voltammetry (LSV) as well as chronoamperometry for the stability and methanol crossover studies. We also present some possible explanations for the relation between the morphological structure and the electrocatalytic behavior of the developed nanocomposites.

## 2. Materials and Methods

### 2.1. Materials and Characterization Methods

**Figure 2:**
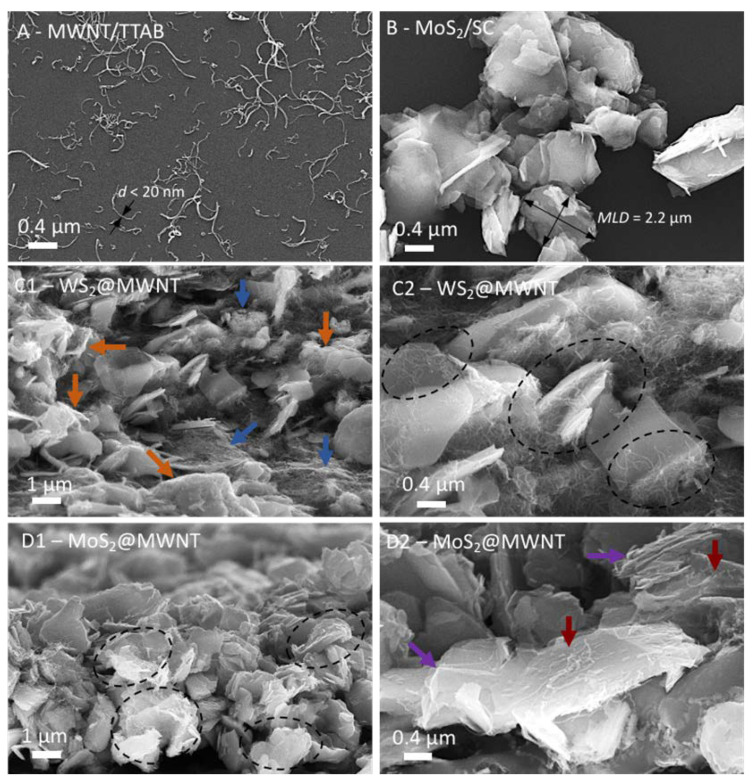
Scanning electron microscopy (SEM) micrographs of the nanocomposite films: (**A**) well-dispersed MWNTs using surfactant TTAB, with arrows showing typical tube widths (<20 nm). (**B**) MoS_2_ particles dispersed by surfactant SC, with the calculation of a typical mean lateral dimension (*MLD*) illustrated. (**C1**,**C2**), WS_2_@MWNT composites, (**D1**,**D2**), MoS_2_@MWNT composites. The arrows and dashed ovals in C1–D2 highlight particular features described in detail in the text.

Multiwalled carbon nanotubes, produced by catalytic chemical vapor deposition and with purity > 95%, were purchased from Cheap Tubes Inc. (Grafton, MA, USA), having outer diameter *d* = 8–15 nm and length *L* = 10–50 μm. Transmission electron microscopy (TEM) imaging of the as-purchased MWNTs confirmed the absence of metal catalyst impurities; see Supplementary Material, Figure S1.1. Furthermore, SEM imaging of the dispersed MWNTs (described in detail below) further confirmed the supplied dimensions and absence of impurities (Figure 2A, and Figure S1.2). WS_2_, MoS_2_, tetradecyltrimethylammonium bromide (TTAB) and sodium cholate hydrate (SC), all with purity ≥ 99%, were acquired from Sigma-Aldrich (St. Louis, MO, USA) and used without further purification. Reagents used for the preparation and performance testing of the electrocatalysts, namely potassium hydroxide (KOH, Riedel-de-Häen, Seelze, Germany), 2-propanol (99.5%, Sigma-Aldrich), Nafion (5 wt% solution in lower aliphatic alcohols and water, Sigma-Aldrich) methanol and 20 wt% Pt/C (HiSPEC^®^ 3000, Alfa Aesar, Haverhill, MA, USA) were used as received. Ultra-pure Milli-Q^®^ (Merck KGaA, Darmstad, Germany) water, with resistivity 18.2 MΩ cm at 25 °C, was used in the preparation of all solutions.

Characterization of the individual surfactant-assisted dispersions of the building blocks, MWNTs and TMDs, was performed by SEM to show the good degree of exfoliation of the materials (Figure 2A,B, and Supplementary Figures S1.2 and S1.3). A FEI Quanta 400FE SEM microscope (Hillsboro, OR, USA) at Centro de Materiais da Universidade do Porto (CEMUP), was used, operating with an electron beam of 25 kV, at different magnifications and secondary electron (SE) mode. Detailed SEM studies were also carried out for the fabricated nanocomposites (Figure 2C1–D2). For imaging of the MWNT and TMD dispersions, the samples were prepared by drop casting 10 µL of each dispersion on a pre-heated silicon wafer (>100 °C, assuring fast solvent evaporation). The fabricated nanocomposite films were fractured in liquid nitrogen, allowing a clean fracture, and the samples were analyzed in a cross-section view for a better visualization of the nanocomposite structure.

### 2.2. Assembly of the Nanocomposite Materials

The assembly process started with the preparation of two dispersions, by surfactant-assisted liquid phase exfoliation, using a previously reported procedure [46]. Briefly, 60 mg of the nanomaterial powder (MWNTs or TMDs) were added to 20 mL of a surfactant aqueous solution (resulting in a 3 mg·mL^−1^ initial loading of the dispersion). The surfactant concentrations used were 5 mmol·kg^−1^ TTAB for MWNTs and 10 mmol·kg^−1^ SC for TMDs. These values of surfactants concentrations were chosen to ensure that maximum dispersibility of each nanomaterial was attained, according to our previous studies with MWNTs [44,45,46] and to recent data on dispersibility of the two TMDs using SC (see Supplementary Figure S1.4a).

Both mixtures were then tip-sonicated, using a Sonics VC 505 with a freshly polished 13 mm tip (500 W, 20 kHz). The vibration amplitude and sonication time were set to 60% and 5 min for MWNTs, and 50% and 23 min for TMDs, as previously optimized [44,45,46]. The total energy transferred per unit mass was 0.20 kJ·mg^−1^ for MWNTs and 0.84 kJ·mg^−1^ for TMDs. An external bath was used to stabilize the temperature of the dispersions. Following sonication, the MWNT dispersions were centrifuged (Centurion Scientific K241R, equipped with a BRK5324 rotor) for 20 min at 4000× *g*, in order to remove impurities (including any residual metal catalyst particles) and large undispersed MWNT agglomerates [46,48,49,50], and the supernatant was collected to build the composites. In the case of the TMD dispersions, it was observed by SEM that the centrifugation step led to a significant reduction of the size of the 2D particles in suspension (mean lateral dimension, *MLD* < 0.3 µm), and since large sheets (typically, *MLD* > 1 µm) were needed to build a well-structured composite, this experimental step was eliminated to build the films (see Supplementary Figure S1.3). Therefore, the final concentration of dispersed TMD nanomaterial corresponds to its initial loading on the samples (since no material is lost to centrifugation). A nominal TMD/MWNT mass ratio of ≈3:1 was used to build the composites; as concerning the negative-to-positive charge ratio (due to the adsorbed surfactants), it is also roughly 3:1 (taking into account that a fraction of the cationic surfactant TTAB in the MWNTs is lost to the sediment due to centrifugation [44]). Overall, this implies net excess of negative charge (owing to the SC-coated TMDs) in the preparation of the nanocomposites, and so the underlying assumption is that basically all the TTAB-coated MWNTs assemble into the composite material.

The individual as-obtained dispersions of the MWNTs and TMDs were then mixed and sonicated together to form the nanocomposites, using the same value of energy per mass used for the MWNTs (0.20 kJ·mg^−1^), to avoid fracture of the nanotubes at higher energy density. After this procedure, the composite samples were vacuum-filtered, rinsed with ethanol and dried overnight.

### 2.3. Evaluation of the Electrocatalytic Activities

A potentiostat/galvanostat PGSTAT 302N (Metrohm Autolab B.V., Utrecht, The Netherlands), controlled by Nova v2.1 software, was used to carry out all electrochemical studies. A conventional three-electrode cell setup was used: a glassy carbon rotating disk electrode (RDE, diameter of 3 mm, Metrohm) as working electrode, a Ag/AgCl (Metrohm, 3 mol·dm^−3^ KCl(aq)) as reference electrode and a carbon rod (Metrohm, diameter of 2 mm) for ORR or a platinum wire (Goodfellow, diameter of 0.6 mm, *l* = 0.5 m, >99.99%) for OER as the counter electrode. All studies were performed at room temperature.

The RDE was conditioned with a polishing process using diamond pastes (Buehler, MetaDI II, Lake Bluff, IL, USA) with particle sizes of 6, 3 and 1 μm, before being modified with the samples. Electrode modification consists of dropping two 2.5 μL droplets of a dispersion containing the materials onto the glassy carbon surface of the RDE, and letting it dry under a constant flux of hot air. The dispersions used to modify the RDE were prepared by mixing the selected nanomaterial (1 mg) with 125 μL of 2-propanol, 125 μL of ultrapure water and 20 μL of Nafion^®^ 117, followed by a 15 min bath ultrasonication (Fisherbrand FB11201, Hampton, VA, USA).

### 2.4. ORR Performance

All ORR studies used KOH aqueous solution (0.1 mol·dm^−3^, 100 mL) saturated with oxygen or nitrogen gas as the electrolyte. To ensure proper gas saturation of the solution, an initial degassing process was done for at least 30 min prior to the study. N_2_-saturated studies served as a blank for the O_2_-saturated ones, and, thus, the current obtained in the former was subtracted from that in the latter. Electrocatalytic performance of the materials toward ORR was studied by cyclic voltammetry (CV) and linear sweep voltammetry (LSV). The scan rate for both was 5 mV·s^−1^, and the rotation speed for LSV was 400, 800, 1200, 1600, 2000 and 3000 rpm.

The *E*_onset_ vs. Ag/AgCl values were converted to *E*_onset_ vs. RHE (reversible hydrogen electrode), using Equation (1):

(1) $$E_{\mathrm{RHE}}=E_{\mathrm{Ag}/\mathrm{AgCl}}+0.059\;\mathrm{pH}+E_{\mathrm{Ag}/\mathrm{AgCl}}^{o}$$

where *E*_RHE_ is potential vs. RHE, *E*_Ag/AgCl_ is potential vs. Ag/AgCl and *E*^o^_Ag/AgCl_ = 0.1976 V (at 25.0 °C).

The onset potential, defined as the potential at which the reduction of oxygen starts, can be determined by different methods [3,51] and is generally assumed as the potential at which the ORR current is 5% of the diffusion-limiting current density. Alternatively, it can be calculated as the potential at which the slope of the voltammogram exceeds a threshold value (*j* = 0.1 mA cm^−2^) [3,51,52]. Here, we considered both methods.

To determine the number of electrons being transferred per O_2_ molecule (*n*_O2_) with LSV data, the Koutecky-Levich (K-L) Equation (2) was used:

(2) $$\frac{1}{j}=\frac{1}{j_{L}}+\frac{1}{j_{k}}=\frac{1}{B\omega^{1/2}}+\frac{1}{j_{k}}$$

where *j* is the measured current density, and *j*_L_ and *j*_k_ are the diffusion-limiting current density and the kinetic current density, respectively. The angular velocity is represented by ω and *B* is related to the diffusion-limiting current density, as shown in Equation (3):

(3) $$B=0.2\;n_{O_{2}}\;F\;{\left(D_{O_{2}}\right)}^{2/3\;}\upsilon^{-1/6}\;C_{O_{2}}$$

where *F* = 96,485 C·mol^−1^, *D*_O_2__ is the O_2_ diffusion coefficient (1.95 × 10^−5^ cm^2^·s^−1^ for this electrolyte), *ν* is the electrolyte kinematic viscosity (8.977 × 10^−3^ cm^2^·s^−1^) and *C*_O_2__ is the bulk concentration of O_2_ (1.15 × 10^−3^ mol·dm^−3^ in this electrolyte). A constant of 0.2 was used, since the rotation speeds are given in rpm.

Methanol resistance was carried out by chronoamperometry in O_2_-saturated KOH for 2500 s, at a fixed potential of *E* = −0.55 V vs. Ag/AgCl and speed rotation or 1600 rpm, where, at 500 s, 2 mL of methanol was added to the electrolyte. Stability tests were conducted by chronoamperometry in O_2_-saturated KOH for 20,000 s, at *E* = −0.55 V vs. Ag/AgCl and 1600 rpm.

### 2.5. OER Performance

OER studies were carried out with an aqueous solution of KOH (0.1 mol·dm^−3^, 100 mL) degassed with oxygen gas. These studies involved acquiring LSV polarization curves from 1.0 to 1.8 V vs. RHE, at a scan rate of 5 mV·s^−1^ and a speed rotation of 1600 rpm. The *i*_R_-compensation (90% of uncompensated resistances, *R*_u_) was applied to all LSV tests where the *R*_u_ values were estimated from *i*-interrupt tests.

## 3. Results and Discussion

### 3.1. Structural Characterization of the Materials by SEM

The morphological features of the 1D and 2D building blocks were characterized by microscopy methods in the bulk pristine state, and after the surfactant-assisted exfoliation and dispersal process (see Supplementary Figures S1.1–1.3), in the light of previous works [44,53]. Representative SEM micrographs of the dispersed MWNTs and TMDs are shown in Figure 2A,B, respectively. Figure 2A shows that after the sonication-centrifugation preparation method, the MWNTs are well-dispersed and individualized in aqueous dispersion by the cationic surfactant TTAB, from the initial bundled agglomerates. Most of the tubes appear isolated (widths of less than 20 nm, consistent with the nominal width provided by the supplier, 8–15 nm) and curvilinear in shape, with lengths of few tenths of nm up to about 2 µm. In Figure 2B, it can be observed that the negatively charged SC-dispersed MoS_2_ particles (sonicated but not centrifuged, as mentioned in Section 2.2), have mean lateral dimensions mostly in the range of 0.5–2 µm (similar results were obtained for WS_2_, processed under exactly the same conditions). Following characterization of the individual building blocks, the prepared WS_2_@MWNT and MoS_2_@MWNT composites were also characterized by SEM, as illustrated in Figure 2C1–D2. For both materials, the images suggest that the 1D and 2D blocks interact, forming tightly bound and mixed composites, as could be expected from the fact that the blocks are coated by surfactants of opposite charge, and hence strong electrostatic interactions in solution are at play (see also Supplementary Figure S1.4b). It is worth mentioning that nanocomposites based on a similar approach, using ionic surfactants and electrostatic interactions as an assembly driving force, have been previously reported [38,54]. Some differences can be seen, however, between the WS_2_@MWNT and the MoS_2_@MWNT materials. Figure 2C1 shows that the WS_2_@MWNT composite is mostly characterized by regions of entangled MWNTs (blue arrows), and embedded and coated WS_2_ 2D particles (orange arrows). Figure 2C2, at higher magnification, reveals further details: some of the TMD particles seem to be deeply embedded in dense networks of MWNTs, with both the basal planes and edges of particles covered by the tubes (as indicated by the dashed ovals).

In contrast, Figure 2D1 and 2D2 show that the MoS_2_@MWNT composite seems to be mostly composed of the 2D particles alternating with horizontally placed MWNTs (dashed ovals), presumably resulting in more compact, stacked layers of the 1D and 2D blocks than the previous material. Figure 2D2, in particular, shows that the nanotubes are lying essentially on the basal planes of the particles (red arrows), with a relatively even separation between them, presumably leaving the TMD edges more exposed to the medium (violet arrows). Whether or not these differences in morphological features between the obtained composites will reflect on their electrocatalytic behavior remained to be seen and was subject to investigation in the next section.

### 3.2. ORR Activity of the Composite Materials

The ORR electrocatalytic performances of pristine WS_2_, WS_2_/SC, MWNT/TTAB, WS2@MWNT, pristine MoS_2_, MoS_2_/SC, centrifuged MoS_2_/SC (MoS_2_/SC w/CF) and MoS_2_@MWNT were initially evaluated by cyclic voltammetry, in N_2_- and O_2_-saturated 0.1 mol·dm^−3^ KOH solution. The results are provided in Supplementary Figure S2.1. In the N_2_-saturated electrolyte solution, none of the studied materials show electrochemical processes in the potential window studied. In contrast, in the O_2_-saturated electrolyte, an ORR peak can be distinguished for all the materials. This peak occurs at *E*_pc_ = 0.58, 0.50, 0.52, 0.58, 0.54, 0.55, 0.55 and 0.72 V vs. RHE for MWNT/TTAB, WS_2_ pristine, WS_2_/SC, WS_2_@MWNT, MoS_2_ pristine, MoS_2_/SC w/CF, MoS_2_/SC and MoS_2_@MWNT, respectively. This confirms the electrocatalytic activity of the materials toward ORR.

**Figure 3:**
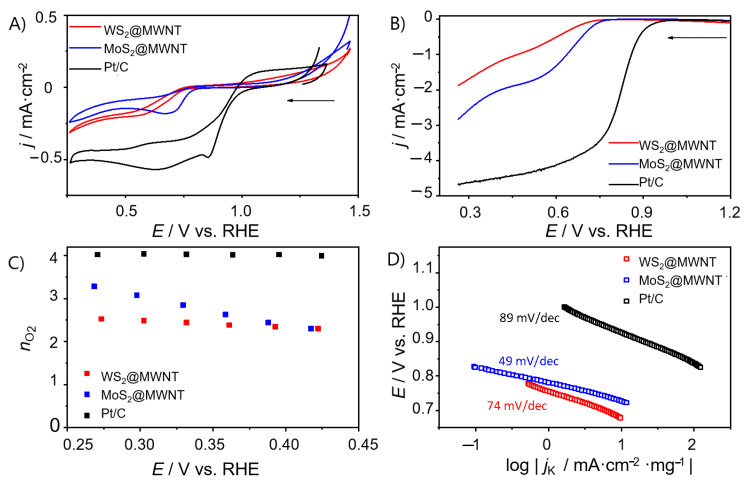
Electrochemical studies on WS2@MWNT, MoS2@MWNT and Pt/C. (**A**) Cyclic voltammograms (CVs) (O_2_-saturated 0.1 mol·dm^−3^ KOH, v = 0.005 V·s^−1^), (**B**) Linear sweep voltammograms (LSVs) at 1600 rpm (O_2_-saturated 0.1 mol·dm^−3^ KOH, v = 0.005 V·s^−1^), (**C**) *n*_O2_ at different potentials, (**D**) Tafel plots.

Figure 3A shows the CVs in O_2_-saturated KOH for WS_2_@MWNT, MoS_2_@MWNT and the benchmark electrocatalyst Pt/C. It can be seen that the obtained results for the nanocomposites are still somewhat inferior compared to that obtained for Pt/C (*E*_pc_ = 0.86 V). Still, there are differences between the composites, with MoS_2_@MWNT showing better performance than WS_2_@MWNT.

To unfold the kinetics of the ORR process at the prepared materials, LSV studies were carried out in a N_2_- and O_2_-saturated electrolyte solution (0.1 mol·dm^−3^ KOH), at different rotation speeds. The LSVs at 1600 rpm for WS_2_@MWNT and MoS_2_@MWNT are presented in Figure 3B. From the LSV curves, onset potential (*E*_onset_), current densities (*j*_L_) and the number of electrons transferred per O_2_ molecule (*n*_O2_) were obtained and are represented in Table 1. The values obtained for MoS_2_@MWNT (*E*_onset_ = 0.73 V vs. RHE and *j*_L_ = −2.74 mA·cm^−2^) are comparable to those obtained for WS_2_@MWNT (*E*_onset_ = 0.71 V vs. RHE and *j*_L_ = −1.87 mA·cm^−2^), however, both are still far from those obtained for the Pt/C electrocatalyst (*E*_onset_ = 0.91 V vs. RHE and *j*_L_ = −4.67 mA·cm^−2^). The differences observed in the *E*_onset_ values are not significant and fall within the experimental uncertainty (<3%).The slightly better performance of MoS_2_@MWNT in terms of *j*_L_ values (uncertainty in *j*_L_ < 7%) may be related to the fact that, in this nanocomposite, the TMD edges are more exposed to the medium, as observed by SEM.

The number of electrons transferred per O_2_ molecule was estimated through Equations (2) and (3). Figure 3C shows the *n*_O2_ values at different potentials for WS_2_@MWNT, MoS_2_@MWNT and Pt/C, while the results for the other materials can be found in the Supplementary Material, Figures S2.2 and S2.3c. WS_2_@MWNT shows a *n*_O2_ value close to 2 electrons, suggesting that the oxygen reduction reaction occurs via the 2-electron indirect mechanism. Nevertheless, the *n*_O2_ values estimated do not vary with the applied potential. For MoS_2_@MWNT, the mean *n*_O2_ value is close to 3 but the potential applied has an impact on the *n*_O2_ values, which decrease as the potential increases. A *n*_O2_ = 3 suggests that the reaction occurs via a mixed 2- and 4-electron mechanism. Although not optimal, since a 4-electron regime was not achieved, these results leave room for improvement.

A possible reason for these results may be related to the particle size of MoS_2_ and WS_2_. According to Li et al. [55], the catalytic activity toward both HER and ORR increased with the decrease in particle size and more importantly, their results showed that selectivity for the 4-electron process may also be related to the Mo edges on the extremely small MoS_2_ nanoparticles (≈2 nm). As referred to in Section 2.2, larger sheets of the TMDs were needed for the construction of the structured composites, justifying the elimination of the centrifugation step. Although, apparently, no significant differences were observed in the electrocatalytic activity of MoS_2_/SC by removing the centrifugation step (Supplementary Figure S2.3), we cannot entirely exclude that in our final materials, the presence of larger particles may affect the ORR activity. Recent work has also shown that different multi-crystalline structures of TMDs, with distinct surface property and electronic performance, greatly impact the materials’ performance in energy storage and conversion, with the metallic phases presenting better results [56]. In our studies, we used the trigonal prismatic structure which, on one hand, is better for exfoliation treatments but, on the other hand, as we find out, leads to worse ORR performance.

Tafel plots, shown in Figure 3D, were obtained from LSV data in Figure 3B at 1600 rpm, in O_2_-saturated KOH. The ORR process exhibits Tafel slopes of 74, 49 and 110 mV·dec^−1^ for WS_2_@MWNT, MoS_2_@MWNT and Pt/C, respectively. These results suggest that for the built nanocomposites, the conversion of MOO^−^ (the intermediate surface-adsorbed species) to MOOH (where M is an empty site on the electrocatalyst surface) rules the global reaction rate, while for Pt/C, it is likely the first discharge step or the consumption of the MOOH species that determines the reaction rate [57].

The ORR performance of the building blocks of the nanocomposites, in various steps of the process, were also studied, and the results are shown in Supplementary Figures S2.2 and S2.3 (data in Supplementary Table S2.1). All TMDs, in the different stages of the process (pristine WS_2_ and MoS_2_, WS_2_/SC, MoS_2_/SC and MoS_2_/SC w/CF), show similar results. This suggests that the presence of the selected surfactants used in this work has little effect on the performance of the materials as electrocatalysts. Nonetheless, in the final step of the assembly process, the nanocomposites were rinsed with ethanol to remove the excess surfactant. Special attention was given to this as, according to recent published works [18,20], the surfactant may play an important role in the electrochemical performance. For example, de-Mello et al. [20] showed that the activity of MoS_2_ towards the HER was enhanced when the surfactant was absent. However, our studies show that presence or absence of surfactant has no impact on the ORR activity.

**Figure 4:**
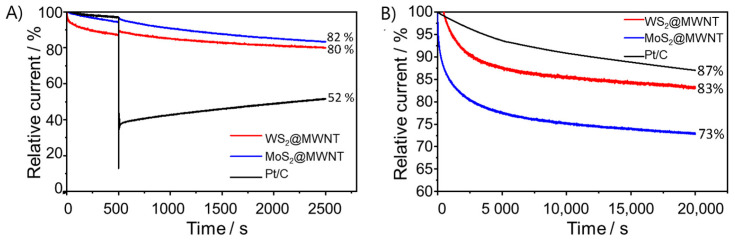
Methanol resistance and stability studies of the WS_2_@MWNT, MoS_2_@MWNT and Pt/C: (**A**) chronoamperometric responses with the addition of 0.5 mol·dm^−3^ methanol (at 500 s) and (**B**) chronoamperometric response at *E* = 0.41 V vs. RHE (O_2_-saturated 0.1 mol·dm^−3^ KOH, and at 1600 rpm) for 20,000 s.

Another relevant parameter that was subject to investigation was the tolerance of the electrocatalysts to methanol crossover. In methanol-based fuel cells, fuel crossover from the anode to the cathode may occur and hence reduce cathodic performance, if electrocatalysts are sensitive to it [58]. As such, tolerance to methanol was evaluated using chronoamperometric tests lasting 2500 s, at 1600 rpm and at *E* = 0.41 V vs. RHE. At the 500 s mark, 2 mL of methanol were injected in the electrolyte (0.1 mol·dm^−3^ KOH). These results are collected in Figure 4A. As it can be observed, Pt/C underwent a decrease in ORR activity of 48%. In contrast, both nanocomposite materials showed better methanol tolerance, with MoS_2_@MWNT retaining 82% of its activity and WS_2_@MWNT 80%. Even though Pt-based materials have better ORR performance than most electrocatalysts, they have the disadvantage of being highly reactive to the methanol oxidation reaction. This affects its ORR activity performance, lowering the obtained current density [3,4]. CV tests were also performed before and after the addition of methanol to further study its effect, and results are depicted in Supplementary Figure S2.4. Once again, it is clear the effect of methanol on the electrocatalytic activity of Pt/C towards ORR in contrast to the little effect on the prepared electrocatalysts.

Long-term stability of the electrocatalysts, another very critical point in the selection of a good electrocatalyst, was also assessed. It was performed by CA during 20,000 s, in O_2_-saturated 0.1 mol·dm^−3^ KOH, at 1600 rpm, and at *E* = 0.41 V vs. RHE, and the obtained results are shown in Figure 4B. After 20,000 s, WS_2_@MWNT retains 83% of its initial current, while MoS_2_@MWNT retains 73%. Even though these values are somewhat lower than that obtained for Pt/C (87%), they suggest good stability of the prepared electrocatalysts.

### 3.3. OER Activity of the Composite Materials

**Figure 5:**
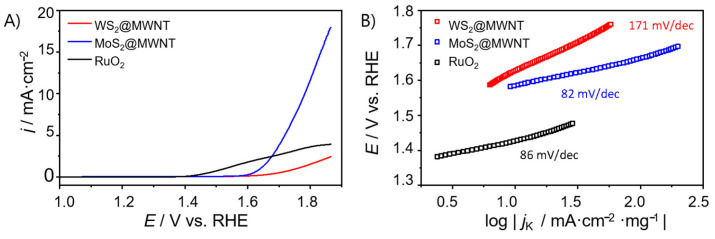
(**A**) OER polarization curves obtained by LSV (O_2_-saturated 0.1 mol·dm^−3^ KOH, *v* = 0.005 V·s^−1^, 1600 rpm) for WS_2_@MWNT, MoS_2_@MWNT and RuO_2_, and (**B**) respective Tafel plots.

The electrocatalytic performance of the nanocomposite materials towards OER was also evaluated. For that, LSV studies were carried out, in a O_2_-saturated 0.1 mol·dm^−3^ KOH electrolyte, at a scan rate of *v* = 0.005 V·s^−1^ and 1600 rpm. The polarization curves obtained are presented in Figure 5. As for ORR, the results were benchmarked using, in this case, one of the state-of-the-art OER electrocatalysts (RuO_2_).

As it can be observed in Figure 5A, WS_2_@MWNT did not present OER activity reaching a value of *j*_max_ of only 2.45 mA·cm^−2^. On the other hand, MoS_2_@MWNT showed good OER activity with a *j*_max_ of 17.96 mA·cm^−2^ and an overpotential of 0.55 V vs. RHE at *j* = 10 mA·cm^−2^. Regarding the benchmark material, RuO_2_, its polarization curves show much lower current density than expected. However, this benchmarking is not completely reliable since the materials compared have different structures and consequently, very different surface areas.

Table 2 gathers the main OER activity parameters, derived from the LSV plots. The values of *j* at *E* = 1.8 V vs. RHE (*j*_1.8_) were also included since neither WS_2_@MWNT nor RuO_2_ reached *j* = 10 mA·cm^−2^.

Like for the ORR studies, the Tafel slopes were determined (Figure 5B) to get an insight into the OER kinetics. Both MoS_2_@MWNT and RuO_2_ presented relatively low values (82 and 86 mV·dec^−1^) when compared with WS_2_@MWNT (171 mV·dec^−1^). The elevated Tafel slope values for OER are characteristic of slow (rate-determining) initial steps, comprising the adsorption of OH- groups on active sites. Therefore, the reduction of the Tafel slope value for the MoS_2_@MWNT in comparison with WS_2_@MWNT indicates that the access of the OH- groups to the active sites is favored in the former nanocomposite.

The building blocks of the nanocomposites (MWNT/TTAB, and pristine and SC-coated WS_2_ and MoS_2_) were also studied for OER (Supplementary Figures S2.5 and S2.6, data in Supplementary Table S2.2). MWNT/TTAB has OER activity, while the pristine TMDs and TMDs with surfactant show poor results. Regarding MoS_2_@MWNT, the nanocomposite has better OER electrocatalytic performance than the sum of its constituents, and hence synergism of properties is suggested.

In short, concerning the electrocatalytic performance of the nanocomposite materials towards the OER, results showed a large difference between them. While MoS_2_@MWNT presented *j*_max_ values of 17.96 mA·cm^−2^ and *η*_10_ = 0.55 V, WS_2_@MWNT only reached current densities of *j*_max_ = 2.45 mA·cm^−2^.

Overall, MoS_2_@MWNT has better electrocatalytic performance than WS_2_@MWNT towards the oxygen reactions. While ORR activity is modest, OER activity is good, suggesting that the nanocomposites may be developed towards bifunctional electrocatalysts, using this fabrication method.

## 4. Conclusions

In this work, nanocomposites of multiwalled carbon nanotubes and TMDs were successfully assembled via a colloidal method based on surfactant-assisted dispersions and electrostatic interactions between oppositely charged surfaces. The final nanocomposite materials were attained in aqueous media, by a simple and cost-effective process that can be easily tuned to adjust the MWNT/TMD ratio. SEM studies showed that, morphologically, the WS_2_@MWNT composite is essentially composed of dense regions of entangled MWNTs with embedded and coated WS_2_ particles, while MoS_2_@MWNT seems to be a tighter composite with MWNTs layers adsorbed horizontally onto to the TMD layers, leaving the edges of the dichalcogenide exposed to the medium.

These materials were then tested as electrocatalysts for both oxygen reactions, showing electrochemical activity towards ORR, with modest performance and good methanol tolerance. The MoS_2_@MWNT nanocomposite had a value of *n*_O2_ close to 3 (indicating a mixed 2- and 4-electron mechanism) and a better overall ORR activity, *E*_onset_ and *j*_L_ values of 0.73 V vs. RHE and −2.74 mA·cm^−2^ respectively, when compared to WS_2_@MWNT (*E*_onset_ = 0.71 V vs. RHE; *j*_L_ = −1.87 mA·cm^−2^). Additionally, MoS_2_@MWNT showed good OER activity, with *η*_10_ and *j*_max_ values of 0.55 V and 17.96 mA·cm^−2^, respectively. These findings point towards potential improvement of the nanocomposites, in order, for instance, to select the best TMD/MWNT combination and develop a good ORR and/or OER electrocatalyst, while having a facile and cost-effective assembly method. Future work will include studies on the role of the TMD/MWNT combination and also the use of hetero-atom-doped MWNTs and other carbon materials, like graphene.

## Figures and Tables

**Table 1:** ORR (oxygen reduction reaction) activity parameters (E_onset_, *j*_L_ and *n*_O2_) for WS_2_@MWNT, MoS_2_@MWNT and Pt/C.

Sample	*E*_onset_/V vs. RHE (5% of *j*)	*E*_onset_/V vs. RHE ^1^ (*j* = 0.1 mA·cm^−2^)	*j*_L_/mA·cm^−2^	*n*_O2_
WS_2_@MWNT	0.71	0.70	−1.87	2.41
MoS_2_@MWNT	0.73	0.74	−2.74	2.87
Pt/C	0.91	0.93	−4.67	4.00

^1^ RHE—reversible hydrogen electrode.

**Table 2:** OER (oxygen evolution reaction) activity parameters (*η*_10_, *j*_max_, and *j*_1.8_) for WS_2_@MWNT, MoS_2_@MWNT and RuO_2_.

Sample	*H*_10_/V (*j* = 10 mA·cm^−2^)	*j*_max_/mA·cm^−2^	*j*_1.8_/mA·cm^−2^
WS_2_@MWNT	-	2.45	1.57
MoS_2_@MWNT	0.55	17.96	11.88
RuO_2_	-	3.94	3.64

## Data Availability

Data is contained within the article or supplementary material.

## Supplementary Materials

The following are available online at https://www.mdpi.com/1996-1944/14/4/896/s1, Section S1 includes additional characterization data. Figure S1.1: representative TEM images of the pristine MWNT powder; Figure S1.2: SEM micrographs of the MWNT/TTAB dispersions at high magnifications. Figure S1.3: SEM imaging of the bulk MoS_2_, after sonication in SC aqueous solution, and after the complete sonication/centrifugation procedure. A Raman spectrum of the latter is also shown. Figure S1.4: dispersibility curves for the TMD/surfactant systems and zeta potential values for the building block particles. In Section S2, further electrochemical data is included. Figure S2.1: CVs of (a) MWNT/TTAB, (b) WS_2_ pristine, (c) WS_2_/SC, (d) WS_2_@MWNT, (e) MoS_2_ pristine, (f) MoS_2_/SC w/CF, (g) MoS_2_/SC, and (h) MoS_2_@MWNT obtained in N_2_ and O_2_-saturated 0.1 mol·dm^−3^ KOH solution, at *v* = 0.005 V·s^−1^. Figure S2.2: electrochemical studies of Pt/C, WS_2_@MWNT nanocomposite, and its building blocks, WS_2_ pristine, WS_2_/SC and MWNT/TTAB. (a) CVs (O_2_-saturated 0.1 mol·dm^−3^ KOH, *v* = 0.005 V·s^−1^), (b) LSVs at 1600 rpm (O_2_-saturated 0.1 mol·dm^−3^ KOH, *v* = 0.005 V·s^−1^), (c) *n*_O2_ at different potentials, (d) Tafel plots. Figure S2.3: electrochemical studies on Pt/C, MoS_2_@MWNT nanocomposite, and its building blocks, MoS_2_ pristine, MoS_2_/SC w/CF, MoS_2_/SC, and MWNT/TTAB. (a) CVs (O_2_-saturated 0.1 mol·dm^−3^ KOH, *v* = 0.005 V·s^−1^), (b) LSVs at 1600 rpm (O_2_-saturated 0.1 mol·dm^−3^ KOH, *v* = 0.005 V·s^−1^), (c) *n*_O2_ at different potentials, (d) Tafel plots. Figure S2.4: methanol resistance studies: (a) chronoamperometric responses of the WS_2_@MWNT, MoS_2_@MWNT and Pt/C materials with the addition of 0.5 mol·dm^−3^ methanol (at 500 s), (b) CV of WS_2_@MWNT before and after methanol addition, (c) CV of MoS_2_@MWNT before and after methanol addition, (d) CV of Pt/C before and after methanol addition. Figure S2.5 presents the OER polarization curves obtained by LSV (O_2_-saturated 0.1 mol·dm^−3^ KOH, *v* = 0.005 V·s^−1^, 1600 rpm) for MWNT/TTAB, WS_2_ pristine, WS_2_/SC, WS_2_@MWNT and RuO_2_; Figure S2.6 shows the OER polarization curves obtained by LSV (O_2_-saturated 0.1 mol·dm^−3^ KOH, *v* = 0.005 V·s^−1^, 1600 rpm) for MWNT/TTAB, MoS_2_ pristine, MoS_2_/SC, MoS_2_@MWNT and RuO_2_. Table S2.1: ORR activity parameters (*E*_onset_, *j*_L_, and *n*_O2_) for MWNT/TTAB, WS_2_ pristine, WS_2_/SC, WS_2_@MWNT, MoS_2_ pristine, MoS_2_/SC w/CF, MoS_2_/SC, and MoS_2_@MWNT. Table S2.2: OER activity parameters (*η*_10_, *j*_max_, and *j*_1.8_) for MWNT/TTAB, WS_2_ pristine, WS_2_/SC, WS_2_@MWNT, MoS_2_ pristine, MoS_2_/SC w/CF, MoS_2_/SC, MoS_2_@MWNT, and RuO_2_.
