# Supplementary material for: Nanocomposites Prepared from Carbon Nanotubes and the Transition Metal Dichalcogenides WS2 and MoS2 via Surfactant-Assisted Dispersions as Electrocatalysts for Oxygen Reactions
Source: Materials (Basel). 2021 Feb 13;14(4):896. doi: 10.3390/ma14040896 (PMC7918728; doi:10.3390/ma14040896)
Supplement: Supplementary file 1 [file materials-14-00896-s001.pdf]

materials

Supplementary Material

Nanocomposites Prepared from Carbon Nanotubes and the Transition Metal Dichalcogenides WS_2 and MoS_2 via Surfactant-assisted Dispersions as Electrocatalysts for Oxygen Reactions

Pedro Ferreira[1,2], Bárbara Abreu[1,2], Cristina Freire[2], Diana M. Fernandes[2*] and Eduardo F. Marques[1]**

1 Centro de Investigação em Química, Departamento de Química e Bioquímica, Faculdade de Ciências, Universidade do Porto 4169-007 Porto, Portugal; up201407953@fc.up.pt (P.F.); barbara.teixeira@fc.up.pt (B.A.)

2 REQUIMTE-LAQV, Departamento de Química e Bioquímica, Faculdade de Ciências, Universidade do Porto 4169-007 Porto, Portugal; acfreire@fc.up.pt

* Correspondence: diana.fernandes@fc.up.pt (D.M.F.); efmarque@fc.up.pt (E.F.M.)

Section S1: Additional data on nanomaterial characterization

S1.1. Purity and exfoliation state of the dispersed materials

The morphology of the purchased MWNTs was evaluated by TEM using a Jeol JEM 1400 with STEM detector. The nanotube powder was mixed in ethanol and deposited on a Cu grid for analysis. TEM imaging allows the identification of catalyst impurities as dark spots in the micrographs, due to the higher atomic weight of these particles, as reported by Loos *et al* [1]. Figure S1.1 (representative TEM micrographs) shows that there is no significant presence of catalyst impurities in the as-received MWNTs used here. Due to the 2D projection of TEM, the few darker regions seen are due to mass-thickness contrast resulting from bending of the MWNTs or superimposed nanotube regions.

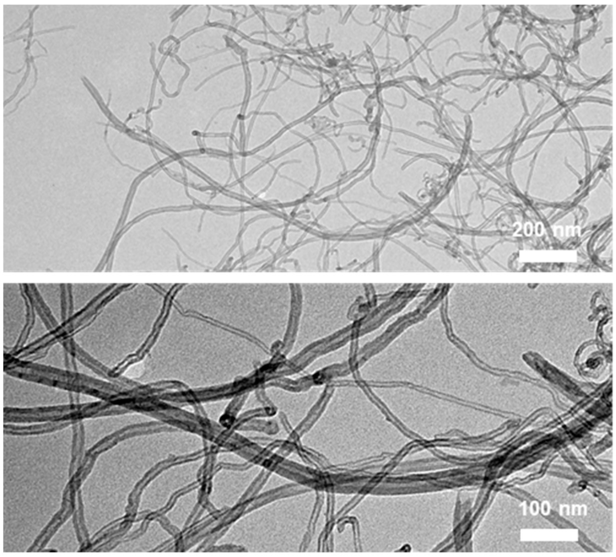

Figure S1.1. Representative TEM micrographs of the commercial MWNTs used in this work.

After dispersion with surfactant TTAB, MWNTs were imaged by SEM (Figure S1.2). The measured MWNT width is typically < 20 nm (consistent with the supplier's range, 8-15 nm) and no trace of metal catalyst particles (bright particles [1]) is seen.

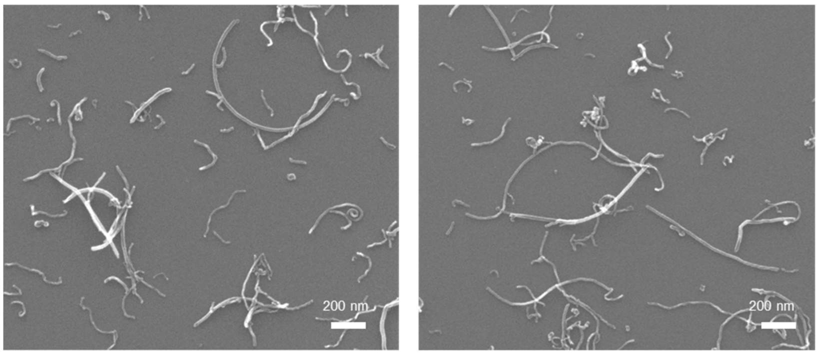

Figure S1.2. Representative SEM micrographs of the MWNT/TTAB dispersions.

Figure S1.3 depicts images of MoS_2 in three different conditions: as-received, after sonication in SC aqueous solution and after the complete sonication/centrifugation procedure described in the experimental section. To evaluate the exfoliation of the TMDs after centrifugation, Raman spectroscopy of the final MoS_2/SC aqueous dispersion was performed on a RAMOS RA532 Raman Analyzer using a laser emitting at 532 nm on glass cuvette, at room temperature. The obtained spectrum is depicted in Figure S1.3 and indicates the presence exfoliated MoS_2, as described in the work of Saito *et al.* and Zhang *et al.* [2,3].

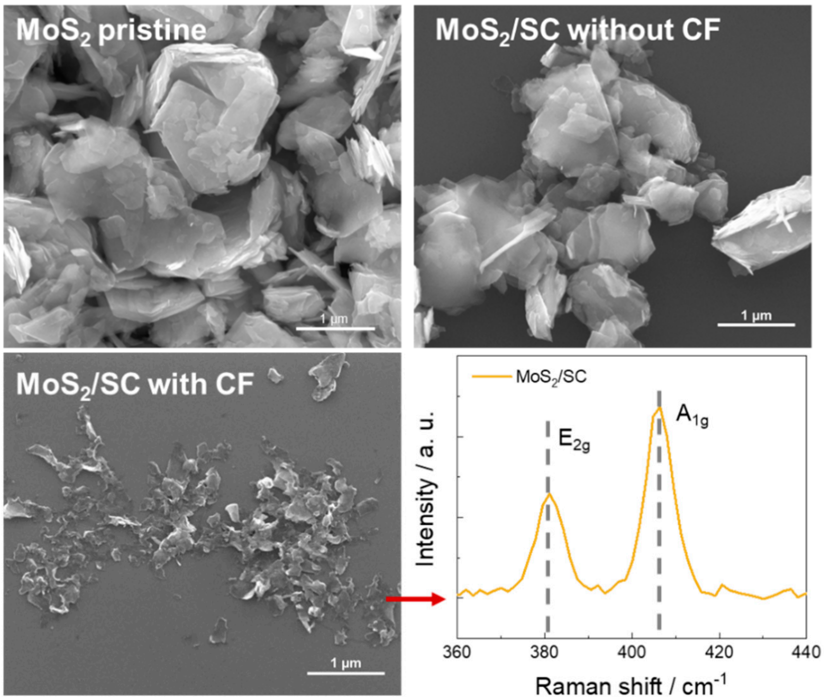

Figure S1.3. Representative SEM micrographs of the pristine MoS_2 and MoS_2/SC dispersions, without centrifugation (CF) and with centrifugation and Raman spectrum of the MoS_2/SC centrifuged sample.

S1.2 Non-covalent functionalization of MWNTs and TMDs

Before the assembly of the nanocomposites, MWNTs and TMDs were physically functionalized using oppositely charged surfactants. To select the optimal surfactant concentration to attain maximum nanomaterial dispersibility, dispersion curves were obtained for all the systems. The MWNT/TTAB curve is reported in [4]. The curves obtained for WS_2 and MoS_2 dispersed with the anionic surfactant SC are shown in Figure S1.4-a). To confirm the non-covalent functionalization of the nanomaterials (i.e. surface charge conferred by the adsorbed surfactant), zeta potential was measured for the dispersions at the selected surfactant concentrations (c_{SC} = 10 mmol·kg^{-1} for WS_2 and MoS_2; c_{TTAB} = 5 mmol·kg^{-1} for MWNTs). The zeta potential was measured at 25 °C using using a LitesizerTM 500 (Anton Paar, Graz, Austria) and DTS 1060C disposable zeta cells.

a)

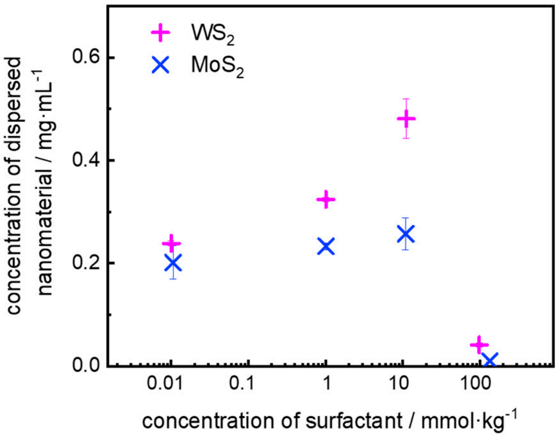

b)

Sample	Zeta potential / mV (*)
WS_2/SC	- 55
MoS_2/SC	- 47
MWNT/TTAB	+ 37

(*) Uncertainty within ± 5%.

Figure S1.4. Dispersion curves obtained for TMDs/SC systems (**a**) and zeta potential determined at the maximum dispersibility point (**b**).

Section S2: Further electrochemical data

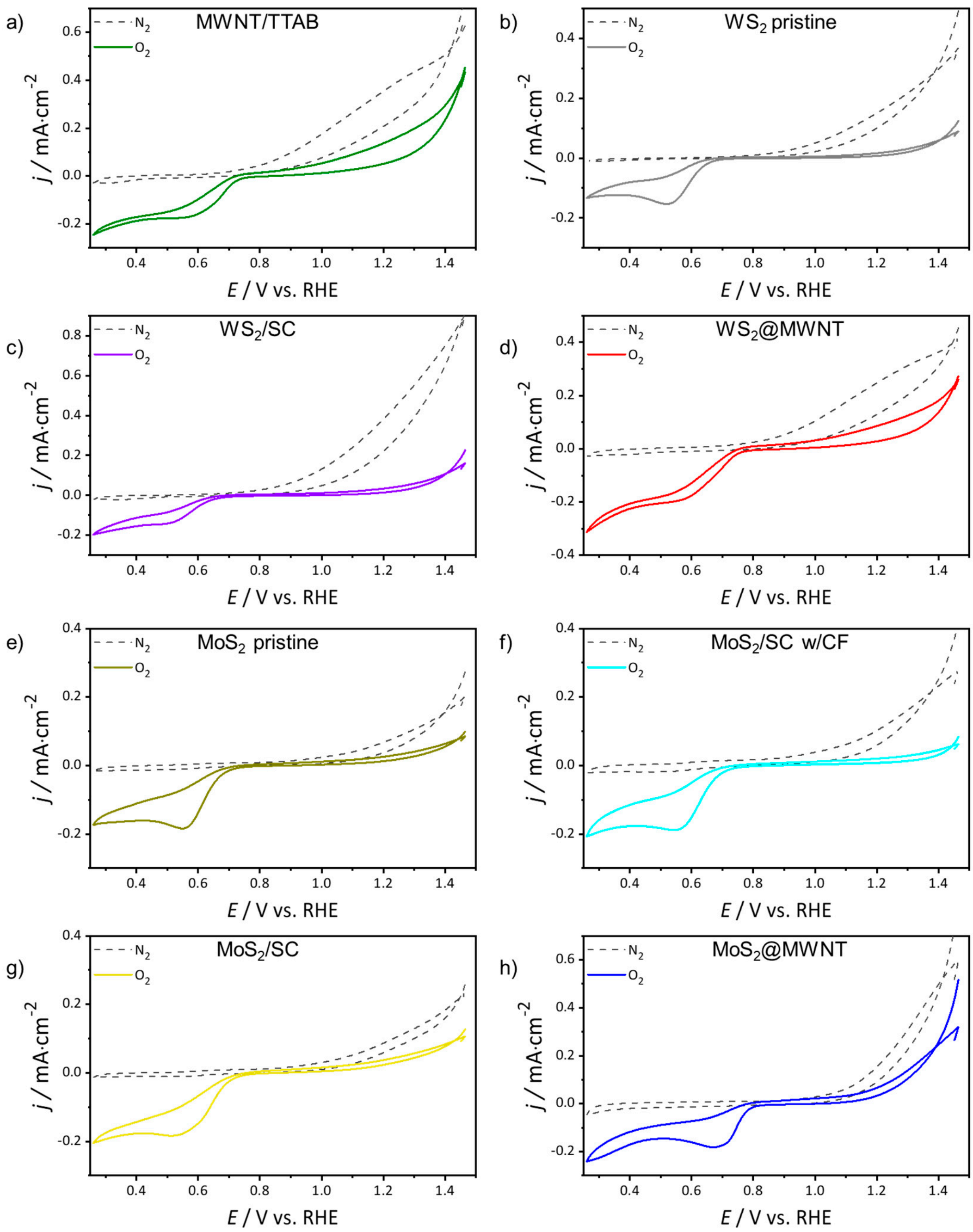

Figure S2.1. CVs of **a**) MWNT/TTAB, **b**) WS_2 pristine, c) WS_2/SC, **d**) WS_2@MWNT, **e**) MoS_2 (pristine), **f**) MoS_2/SC w/CF, **g**) MoS_2/SC (without CF), and **h**) MoS_2@MWNT obtained in N_2- (dashed line) and O_2-saturated (full line) 0.1 mol·dm^{-3} KOH solution, at v = 0.005 V·s^{-1}.

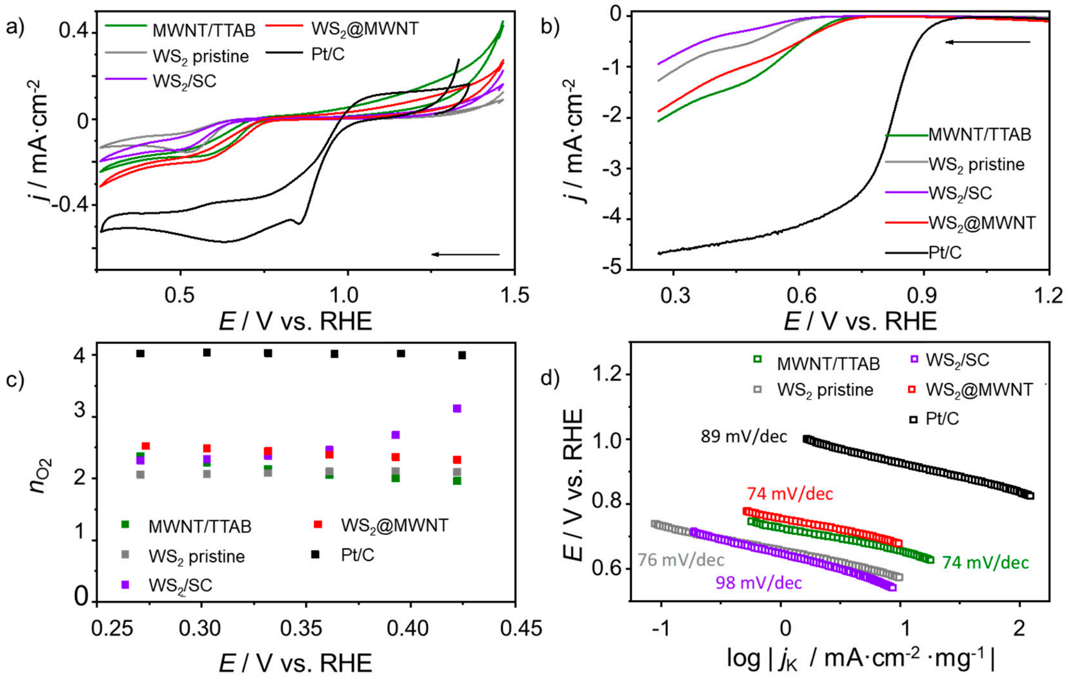

Figure S2.2. Electrochemical studies of Pt/C, WS_2@MWNT nanocomposite, and its building blocks, WS_2 (pristine), WS_2/SC and MWNT/TTAB: **a**) CVs (O_2-saturated 0.1 mol·dm^{-3} KOH, v = 0.005 V·s^{-1}); **b**) LSVs at 1600 rpm (O_2-saturated 0.1 mol·dm^{-3} KOH, v = 0.005 V·s^{-1}); **c**) n_{O2} at different potentials; **d**) Tafel plots.

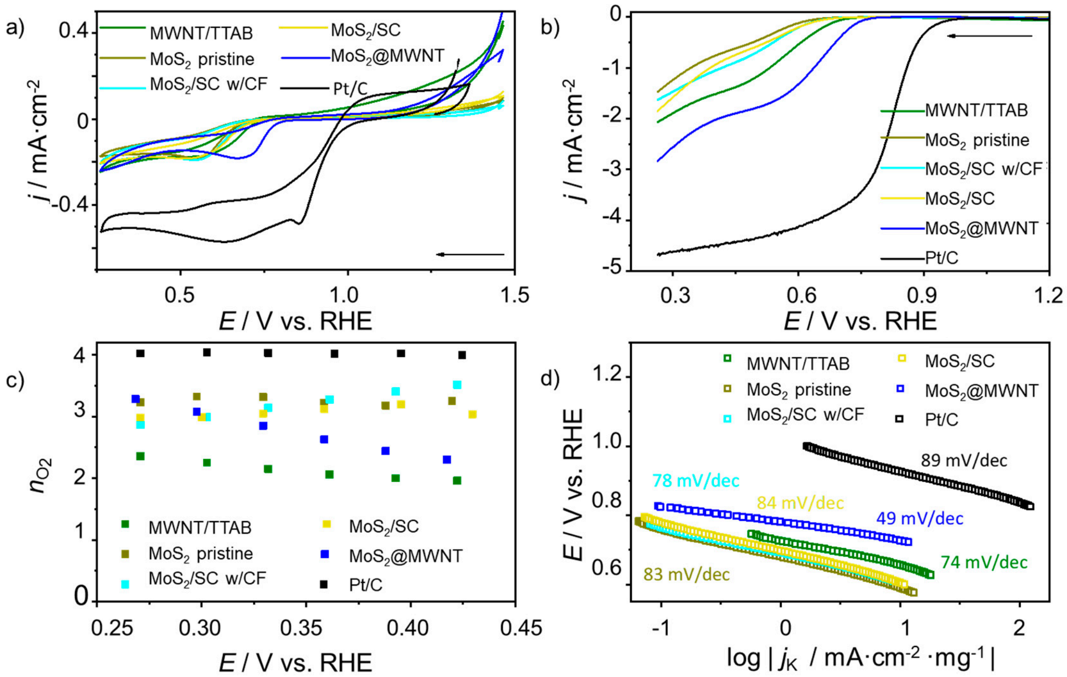

Figure S2.3. Electrochemical studies of Pt/C, MoS_2@MWNT nanocomposite, and its building blocks, MoS_2 (pristine), MoS_2/SC w/CF, MoS_2/SC (without CF), and MWNT/TTAB: **a**) CVs (O_2-saturated 0.1 mol·dm^{-3} KOH, v = 0.005 V·s^{-1}); **b**) LSVs at 1600 rpm (O_2-saturated 0.1 mol·dm^{-3} KOH, v = 0.005 V·s^{-1}); c) n_{O2} at different potentials; d) Tafel plots.

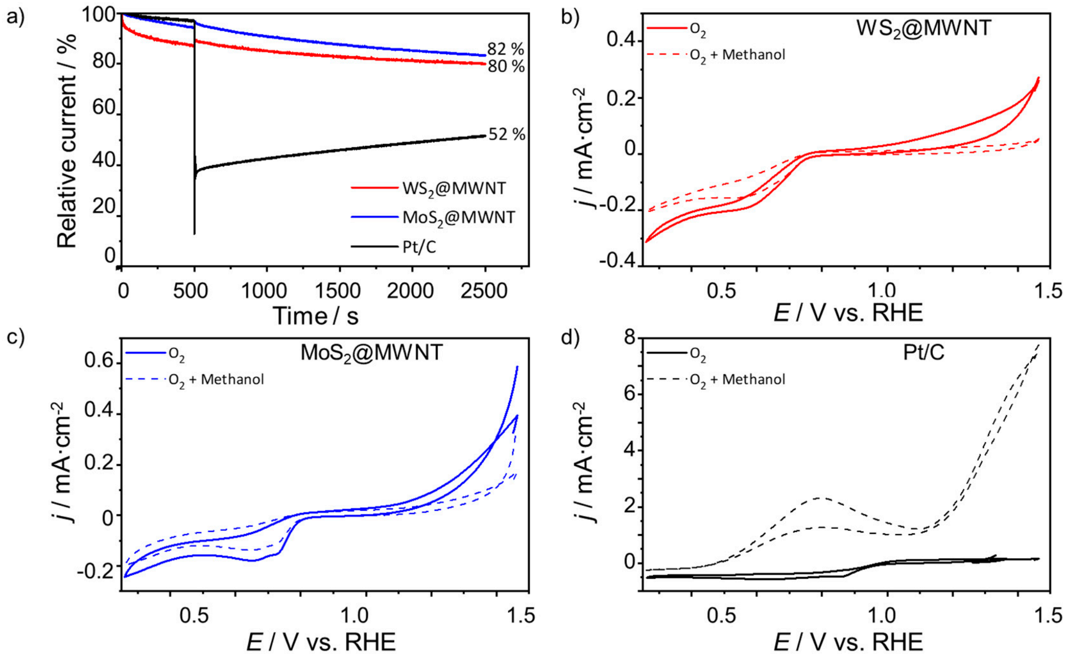

Figure S2.4. Methanol resistance studies: **a**) chronoamperometric responses of the WS_2@MWNT, MoS_2@MWNT and Pt/C materials with the addition of 0.5 mol·dm^{-3} methanol (at 500 s); **b**) CV of WS_2@MWNT before and after methanol addition; **c**) CV of MoS_2@MWNT before and after methanol addition; **d**) CV of Pt/C before and after methanol addition.

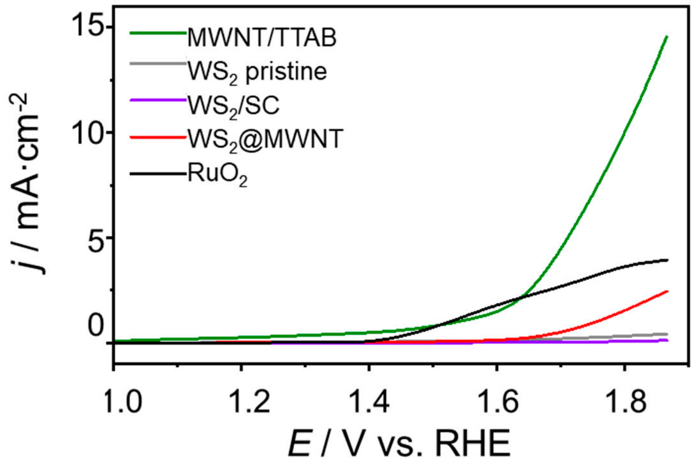

Figure S2.5. OER polarization curves obtained by LSV (O_2-saturated 0.1 mol·dm^{-3} KOH, v = 0.005 V·s^{-1}, 1600 rpm) for MWNT/TTAB, WS_2 (pristine), WS_2/SC, WS_2@MWNT and RuO_2.

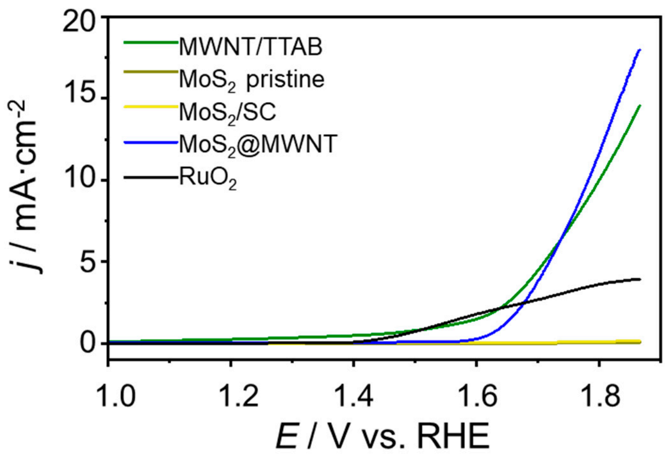

Figure S2.6. OER polarization curves obtained by LSV (O_2-saturated 0.1 mol·dm^{-3} KOH, v = 0.005 V·s^{-1}, 1600 rpm) for MWNT/TTAB, MoS_2 (pristine), MoS_2/SC, MoS_2@MWNT and RuO_2.

Table S2.1. ORR activity parameters (E_{onset}, j_L, and n_{O2}) for MWNT/TTAB, WS_2 pristine, WS_2/SC, WS_2@MWNT, MoS_2 (pristine), MoS_2/SC w/CF, MoS_2/SC, and MoS_2@MWNT.

Sample	E_{onset} / V vs. RHE (5% of j_{max})	E_{onset} / V vs. RHE (j = 0.1 mA·cm^{-2})	j_L / mA·cm^{-2}	n_{O_2}
MWNT/TTAB	0.68	0.68	−2.07	2.13
WS_2 pristine	0.62	0.60	−1.27	2.09
WS_2/SC	0.61	0.57	−0.94	2.36
WS_2@MWNT	0.71	0.70	−1.87	2.41
MoS_2 pristine	0.63	0.62	−1.63	3.25
MoS_2/SC w/CF	0.64	0.63	−1.51	3.20
MoS_2/SC	0.64	0.63	−1.90	2.81
MoS_2@MWNT	0.73	0.74	−2.74	2.87

Table S2.2. OER activity parameters (η_{10}, j_{max}, and $j_{1.8}$) for MWNT/TTAB, WS_2 pristine, WS_2/SC, WS_2@MWNT, MoS_2 pristine, MoS_2/SC w/CF, MoS_2/SC, MoS_2@MWNT, and RuO_2.

Sample	η_{10} / V (j = 10 mA·cm^{-2})	j_{max} / mA·cm^{-2}	$j_{1.8}$ / mA·cm^{-2}
MWNT/TTAB	0.59	14.55	10.15
WS_2 pristine	-	0.42	0.32
WS_2/SC	-	0.12	0.076
WS_2@MWNT	-	2.45	1.57
MoS_2 pristine	-	0.071	0.039
MoS_2/SC	-	0.17	0.12
MoS_2@MWNT	0.55	17.96	11.88
RuO_2	-	3.94	3.64

References

1. Loos, J.; Grossiord, N.; Koning, C.E.; Regev, O. On the fate of carbon nanotubes: Morphological characterisations. *Compos. Sci. Technol.* **2007**, *67*, 783–788.
2. Saito, R.; Tatsumi, Y.; Huang, S.; Ling, X.; Dresselhaus, M.S. Raman spectroscopy of transition metal dichalcogenides. *J. Phys. Condens. Matter* **2016**, *28*, 353002.
3. Zhang, X.; Qiao, X.-F.; Shi, W.; Wu, J.-B.; Jiang, D.-S.; Tan, P. Phonon and Raman scattering of two-dimensional transition metal dichalcogenides from monolayer, multilayer to bulk material. *Chem. Soc. Rev.* **2015**, *44*, 2757–2785, doi:10.1039/c4cs00282b.

4. Abreu, B.; Rocha, J.; Fernandes, R.M.; Regev, O.; Furó, I.; Marques, E.F. Gemini surfactants as efficient dispersants of multiwalled carbon nanotubes: Interplay of molecular parameters on nanotube dispersibility and debundling. *J. Colloid Interface Sci.* **2019**, *547*, 69–77.
